# Supplementary material for: Interest on reserves, helicopter money and new monetary policy
Source: PLoS One. 2021 Jul 19;16(7):e0253956. doi: 10.1371/journal.pone.0253956 (PMC8289106; doi:10.1371/journal.pone.0253956)
Supplement: Attachment — Submitted filename: Response to Referee Report 2.pdf [file pone.0253956.s001.pdf]

Response to Reviewer 2 on "Interest on reserves, helicopter money and new monetary policy" for *PLOS ONE*

I thank the referee for a positive feedback and providing thoughtful and fair comments on it. Below I will outline how I would respond to the referees' suggestions:

1. "I found section 2 to be very confusing. I actually think the paper is easier to understand if section 2 is removed and the reader goes straight to section 3. This has the advantage of avoiding repeating equations as in the current version. Any important content in section 2 can be incorporated in section 3.

Remove Tables 1 to 6. I found them confusing and not very informative".

My Response: I separate section 2 from section 3 to separate the environment of the model and each agent's optimal problem. If my paper followed a standard New Keynesian model, Section 2 would not be necessary. However, transactions with reserves and deposits are not dense in the literature. I hope that my paper will be easily understood for all non-experts, so I trade off between the paper length and details of the environment.

I insert the Tables 1 to 6 for the same reason. Tables 1 to 6 are unnecessary for economists working at central banks or economists specializing in monetary policy. However, many economists who do not fall into the above categories could find them useful. In the case that the editor agrees with the referee, I am happy to move them to the Appendix.

2. "I found the calibration of loan amortization (δb) odd. In section 2.4 it is said that if an agent borrows £1 then the repayment will be:
δ_b in year 1
δ_b x (1-δ_b) in year 2
δ_b x 〖(1-δ_b)〗 ^2 in year 3
and so on…
If one sums the numbers from year 2 onwards then we get:
Sum=δ_b×(δ_b)/((1-δ_b))
With δ_b set at 0.5 then we get the above is 0.5.
Even if we were to add a discount factor to the above (say β) it would not change much.
Compare this to a perpetuity which has present value of 1/r. If we set r=0.04 then we get 25.
The loan amortization seems to me to be implying an excessively high

interest rate (or maybe there is something I am missing here…). I would therefore suggest adopting a different value for δ_b".

My Response: The nominal sum of loan payment will be equal to 1 if the nominal amount of borrowing is 1:

$$\delta^b + \delta^b(1-\delta^b) + \delta^b(1-\delta^b)^2 + \cdots. + \delta^b(1-\delta^b)^n + \cdots.$$
$$= \delta^b[1 + (1-\delta^b) + (1-\delta^b)^2 + \cdots. + (1-\delta^b)^n + \cdots]$$
$$= \frac{\delta_b}{1-(1-\delta^b\ )}$$
$$= 1$$

Furthermore, the price of loan q^l (<1 in the steady state). When banks give loans to households, the nominal amount of loan is \$1, but households only receive \$ q^l. If we calculate the interest of the loan, it will be equal to r^h (equation 12), which is equal around to 4.25% in the steady state.

3. "The paper is fairly well written in the sense that it is clear about what it does. But there are many grammar mistakes. I would suggest having it proof-read".

My Response: I tried fixing grammar mistakes as much as possible in my paper. I also asked one of my colleagues in English department to help me. Hopefully, this revised version could reduce the errors to the minimum level.
